# Is prolonged intermittent renal replacement therapy actually safe for hemodynamically unstable patients

**DOI:** 10.1186/s13054-023-04438-1

**Published:** 2023-04-21

**Authors:** Patrick M. Honore, Sydney Blackman, Emily Perriens, Ibrahim Bousbiat, Min-Min Wang

**Affiliations:** 1 UCLouvain Medical School, CHU UCL Godinne Namur, Avenue G Thérasse 1, 5530 Yvoir, Belgium; 2 Brugmann University Hospital, ULB University, Brussels, Belgium; 3 Acute Therapies Greater China, Baxter International Inc., Shanghai, China

**Keywords:** PIRRT, Hemodynamic stability, Controversy, Small amount of acetate

Routine use of prolonged intermittent renal replacement therapy (PIRRT) in New Zealand and Canada is long established [1]. According to Clark et al. [1], it is a cost-effective technique when compared to continuous renal replacement therapy (CRRT) and safely provides RRT for hemodynamically unstable patients. However, outcomes from other studies concerning hemodynamic stability tend to differ. Sustained low efficiency dialysis (SLED) (A form of PIRRT) is an effective treatment for acute kidney injury (AKI) [2]. However, its hemodynamic safety is controversial [2]. An high frequency of hypotension during SLED has been reported, leading to dialysis termination in up to 11% of cases [2]. Cardiovascular instability during intermittent hemodialysis (IHD) or SLED may be due to the acetate used as the pH-stabilizing factor in standard bicarbonate dialysate (SBD) (3–7 mM) [2]. Studies in patients with end-stage renal disease (ESRD) have shown that even quantities as low as 3 mM of acetate in SBD can cause considerable intradialytic acetatemia with blood acetate accumulation (BAA) as high as 12 times normal values [2]. Thus, IHD with SBD is associated with more frequent hypotension than IHD with acetate-free dialysate (AFD) [2]. Researchers have established that half of the patients had pre-dialysis BAA [2]. However, during SLED with AFD, patients’ levels of acetate did not change significantly [2]. This may explain the decreased of acetate metabolism in certain categories of AKI after cardiovascular surgery (CS) [2]. Their results are generally consistent with existing evidence obtained in patients with ESRD during IHD with SBD, where acetate increased levels were reported [3]. Cardiac depression induced by acetate has been studied in ESRD. It is assumed that BAA induces hypoxia, with the activation of proinflammatory cytokines [4]. Acetate also activates nitric oxide which causes vasodilatation [4]. The clinical manifestation of the BAA is an arterial pressure decrease and a heart rate increase [5]. Their study has shown that certain categories of patients with AKI after CS have lower rates of acetate metabolism [2]. Clearly PIRRT is not completely safe, especially in the context of SLED using SBD leading to high values of acetatemia and risks of hemodynamic complications [2]. Replacing acetate by hydrochloric acid in SBD is a promising alternative; however, it is only done in a minority of cases [2]. Acetate is widely used as a weak acid acting as a dialysate buffer, thus avoiding this precipitation [5]. Even at the low concentrations (3–4 mmol/L) used, it reaches blood levels higher than physiological [5], leading to undesirable outcomes by poorly understood mechanisms [5].

## Data Availability

Not applicable.

## Abbreviations

PIRRT: Prolonged intermittent renal replacement therapy
CRRT: Continuous renal replacement therapy
SLED: Sustained low efficiency dialysis
AKI: Acute kidney injury
IHD: Intermittent hemodialysis
ESRD: End-stage renal disease
SBD: Standard bicarbonate dialysate
AFD: Acetate-free dialysate
BAA: Blood acetate accumulation
CS: Cardiovascular surgery
